# Formononetin, a novel FGFR2 inhibitor, potently inhibits angiogenesis and tumor growth in preclinical models

**DOI:** 10.18632/oncotarget.6310

**Published:** 2015-11-12

**Authors:** Xiao Yu Wu, Hao Xu, Zhen Feng Wu, Che Chen, Jia Yun Liu, Guan Nan Wu, Xue Quan Yao, Fu Kun Liu, Gang Li, Liang Shen

**Affiliations:** ^1^ Department of Surgical Oncology, Affiliated Hospital of Nanjing University of Traditional Chinese Medicine, Nanjing, China; ^2^ Division of Gastrointestinal Surgery, Department of General Surgery, The First Affiliated Hospital of Nanjing Medical University, Nanjing, China; ^3^ Department of General Surgery, Jiangsu Cancer Hospital, The Affiliated Cancer Hospital of Nanjing Medical University, Nanjing, China; ^4^ Laboratory of Biotechnology and Biological Resource Utilization in Universities of Shandong, College of Life Science, Dezhou University, Dezhou, Shandong Province, China

**Keywords:** formononetin, angiogenesis, breast cancer, FGFR2, Akt

## Abstract

Most anti-angiogenic therapies currently being evaluated in clinical trials target vascular endothelial growth factor (VEGF) pathway, however, the tumor vasculature can acquire resistance to VEGF-targeted therapy by shifting to other angiogenesis mechanisms. Therefore, other potential therapeutic agents that block non-VEGF angiogenic pathways need to be evaluated. Here we identified formononetin as a novel agent with potential anti-angiogenic and anti-cancer activities. Formononetin demonstrated inhibition of endothelial cell proliferation, migration, and tube formation in response to basic fibroblast growth factor 2 (FGF2). In *ex vivo* and *in vivo* angiogenesis assays, formononetin suppressed FGF2-induced microvessel sprouting of rat aortic rings and angiogenesis. To understand the underlying molecular basis, we examined the effects of formononetin on different molecular components in treated endothelial cell, and found that formononetin suppressed FGF2-triggered activation of FGFR2 and protein kinase B (Akt) signaling. Moreover, formononetin directly inhibited proliferation and blocked the oncogenic signaling pathways in breast cancer cell. *In vivo*, using xenograft models of breast cancer, formononetin showed growth-inhibitory activity associated with inhibition of tumor angiogenesis. Moreover, formononetin enhanced the effect of VEGFR2 inhibitor sunitinib on tumor growth inhibition. Taken together, our results indicate that formononetin targets the FGFR2-mediated Akt signaling pathway, leading to the suppression of tumor growth and angiogenesis.

## INTRODUCTION

Tumor angiogenesis is essential for the development and progression of malignant tumors [1]. Although many putative regulators of angiogenesis have been identified, vascular endothelial growth factor (VEGF) has been particularly strongly implicated in tumor-associated angiogenesis [2]. Vascular endothelial growth factor receptor 2 (VEGFR2) is the major effecter for execution of VEGF-stimulated cell proliferation, vascular permeability, cell migration, and cell survival, leading to angiogenesis. Antagonizing angiogenesis-related receptor tyrosine kinase (RTK) is a promising therapeutic strategy in oncology. A number of small molecule VEGFR2 inhibitors have been reported, including sunitinib, sorafenib, and vandetanib [3]. However, other angiogenic regulatory factors switch on during cancer progression and induce resistance to existing antiangiogenic therapy [4]. Besides VEGF, There is a family of proteins that include placenta growth factor (PIGF), fibroblast growth factor (FGF1), FGF2, Fms-like tyrosine kinase 3 (Flt3), c-Met, and platelet-derived growth factor receptor-alpha (PDGFRα) directly participate in the genesis of blood capillaries and lymphatic vessels [5]. Furthermore, recent studies have identified FGF2 as a direct activator of phosphatidylinositol-4, 5-bisphosphate 3-kinase (PI3K)-protein kinase B (Akt), which are key stimuli known to initiate endothelial cell migration, invasion and differentiation. Recent studies have suggested that the PI3K might play a vital role in tumor angiogenesis [6]. Akt is a pivotal downstream target of PI3K during angiogenesis. Akt regulates multiple cellular processes including tumor angiogenesis, cell cycle progression, cell growth, cell migration, and cell metabolism [7]. Fbroblast growth factor receptor 2 (FGFR2) activation after FGF2 binding causes phosphorylation of Akt signaling resulting in increased activation of signal transducer and activator of transcription 3 (STAT3), c-Jun and nuclear factor kappa-light-chain-enhancer of activated B cells (NF-κB) p65 [8]. STAT3 is often constitutively active in many human cancer cells, including multiple myeloma, leukemia, lymphoma, and solid tumors. STAT3 is a latent transcription factor that resides in the cytoplasm. Upon activation, STAT3 dimerizes, translocates to the nucleus and binds to nuclear DNA to modulate transcription of target genes. The activation of STAT3 results in expression of many target genes including matrix metalloproteinases (MMPs), cyclooxygenase-2 (COX-2) and angiopoietin-2 (Ang-2) which are required for tumor cell migration, angiogenesis as well as metastasis [9].

Currently, several strategies have been already reported to block the action of kinase signialing pathway besides VEGF-VEGFR2, including natural compounds, peptidomimetic compounds, and small molecules. Phytochemicals are potential novel leads for developing anti-angiogenic drugs [10]. Flavonoids are polyphenolic substances, widely distributed in almost every food plant, that possess antiviral, antimicrobial, anti-inflammatory, anti-thrombotic, antineoplasic, antimutagenic, and cytoprotective effects on different cell types [11]. The dried root of Astragalus membranaceus (Radix Astragali) has a long history of medicinal use in traditional chinese medicine as an immunomodulating agent in mixed herbal decoctions to treat the diarrhea, common cold, anorexia and fatigue [12]. In contemporary pharmacotherapy, Radix Astragali has been used to ameliorate the side-effects of cytotoxic antineoplastic drugs [13]. Formononetin is one of the major isoflavonoid constituents isolated from Astragalus membranaceus and has been demonstrated diverse pharmacological benefits [14]. It possesses anti-angiogenic activity in human colon cancer cells and tumor xenograft. Formononetin also promotes cell cycle arrest via downregulation of Akt/Cyclin D1/CDK4 in human prostate cancer cells [14]. Nevertheless, this novel compound has also been shown to suppress the proliferation of human non-small cell lung cancer through induction of cell cycle arrest and apoptosis [15]. However, data on the influence of formononetin on breast cancer angiogenesis and the underlying mechanisms are yet to be fully elucidated. Despite important progress in adjuvant and neoadjuvant therapies, angiogenesis often develops in breast cancer patients and remains the leading cause of their deaths. Recently, small-molecule multikinase inhibitors targeting VEGFRs have been shown to have therapeutic potential in preclinical and/or clinical testing against breast tumour. For example, sorafenib, which can inhibit VEGFRs, has been used successfully in the clinic to prolong the survival rate of hepatocarcinoma patients. However, quite a few multi-target therapies show toxicity and have only moderate response rates. In the present study, we investigate the effects of formononetin on angiogenesis and the growth of human breast cancer cells and nude mouse xenografts. The results obtained provide evidence for the broader use of formononetin as an anti-angiogenesis agent against human breast cancers.

In the present study, we described formononetin inhibited FGF2 induced FGFR2 activation at relatively low concentrations *in vitro* assays. Based on its molecular mechanism, it significantly inhibited endothelial cells proliferation, migration, invasion, and tube formation. Moreover, it exhibited the ability to inhibit angiogenesis in the rat artic ring assay and chick embryo chorioallantoic membrane (CAM) angiogenesis model. At molecular level we showed that formononetin inhibited angiogenesis by blocking the FGFR2-mediated PI3K-Akt signaling pathways in endothelial cells. Because of the critical role of STAT3 activation in endothelial cells migration and tube formation, we hypothesized that formononetin may mediate its effects through suppression of STAT3 activity. *In vitro*, we found that formononetin indeed suppressed FGF2 inducible STAT3 activation. Additional, formononetin directly inhibited breast cancer cell proliferation and blocked the oncogenic PI3k-Akt signaling pathways in tumor cells. Furthermore, this compound had excellent pharmacokinetic profiles that made it suitable for chronic once-daily oral administration *in vivo*. Formononetin significantly inhibited the growth of tumor xenografts in athymic mice. In additional, we investigated the effect of formononetin and sunitinib (a RTK inhibitor targeting VEGFR2) combination treatment in cancer cells. We found that the combination treatment significantly decreased cancer cell invasion stimulated by FGF2 *in vitro* and tumor growth *in vivo*. Taken together, our data suggested that formononetin could function as a novel FGFR2 inhibitor that suppresses tumor angiogenesis and growth.

## RESULTS

### Kinase inhibition profile of formononetin

**Figure 1:**
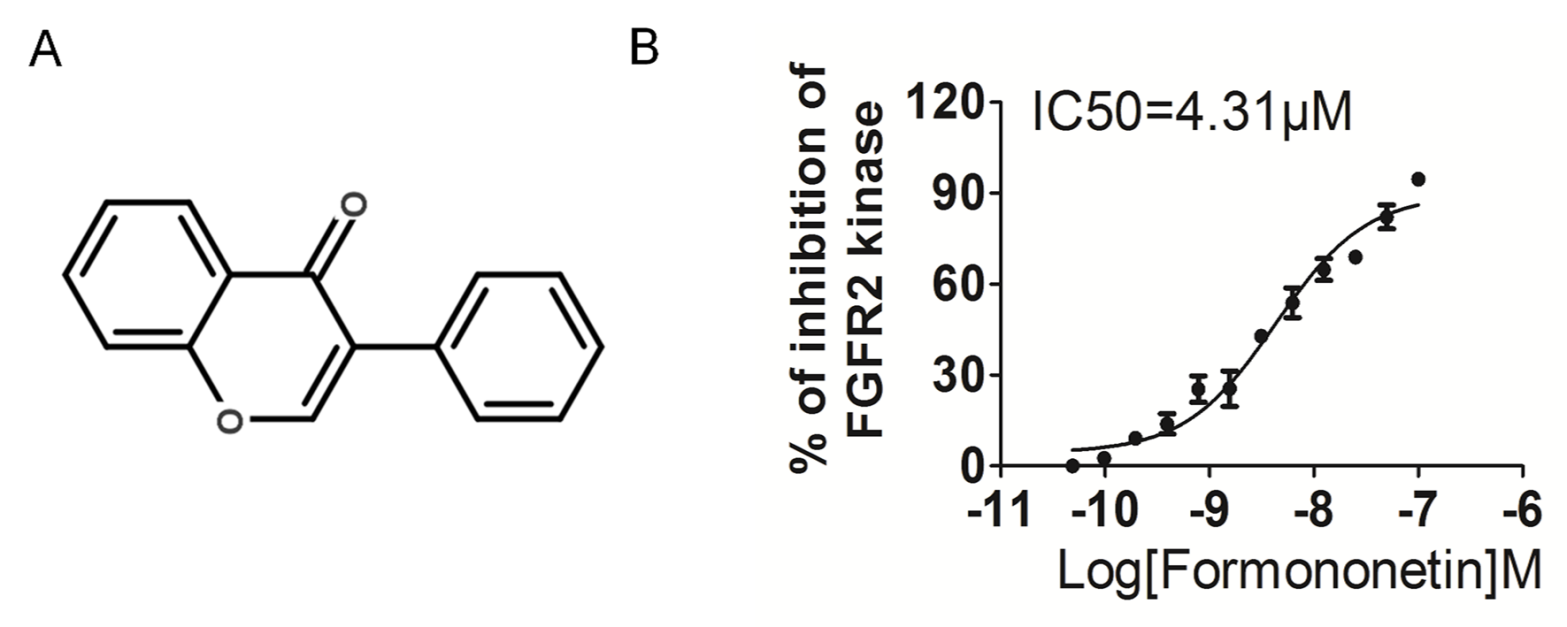
Formononetin decreases FGFR2 kinase activity **A.** Chemical Structure of formononetin. **B.** Formononetin inhibited FGFR2 kinase activity *in vitro*. Data are from three independent experiments and are mean ± SD.

In this study, formononetin was screened by kinase inhibition assay by the use of radiometric assays provided by Kinase Profile Service (Millipore, UK). The effects of formononetin (Figure 1A) on kinase activity were detected using the scintillation proximity assay method at an enzymatic level. As shown in Table 1, formononetin exhibited great inhibitory activity on FGFR2 with an inhibitory rate of 89% at 1 μM. In addition, the inhibitory activity of formononetin was examined against FGFR1 because of its structural and expression level similarity to the FGFR2. Formononetin showed a relatively low inhibitory rate of 57%, 5%, 0%, and 1% against FGFR1, VEGFR2, PDGFRα, and PDGFRβ at 1 μM, respectively. Moreover, excellent selectivity for FGFR2 was evident compared with a range of unrelated tyrosine and serine/threonine kinases, including Flt3, c-Kit, c-Met, epidermal growth factor receptor (EGFR), c-RAF etc.

**Table 1:** *In vitro* profile of formononetin against a panel of 20 kinases

Kinase	Inhibition rate at 1 μM (%)
FGFR2	89 ± 2
FGFR1	57 ± 4
VEGFR2	5 ± 0
Flt3	7 ± 2
PDGFRα	0 ± 2
PDGFRβ	1 ± 6
c-Kit	4 ± 2
Haspin	−1 ± 5
Aurora-A	5 ± 2
ErbB4	2 ± 2
IKKβ	−4 ± 4
c-Met	9 ± 6
CDK2	−11 ± 7
EGFR	19 ± 4
PI3K	1 ± 2
JNK	−7 ± 2
mTOR	4 ± 1
GSK3β	0 ± 0
JAK	12 ± 6
c-RAF	4 ± 0

The assays were performed in two independent experiments. Data are means ± SD

To investigate whether formononetin decreased the kinase activity of FGFR2, we performed *in vitro* kinase assay with different concentrations of formononetin using CycLex^®^ FGFR2 Kinase Assay kit according to manufacturer suggested methods. Our data demonstrated that formononetin directly inhibited FGFR2 kinase activity in a dose-dependent manner with an IC50 of ~4.31 μM (Figure 1B). All these results indicated that formononetin was a potent FGFR2 inhibitor.

### Formononetin inhibited the response of HUVECs to FGF2

**Figure 2:**
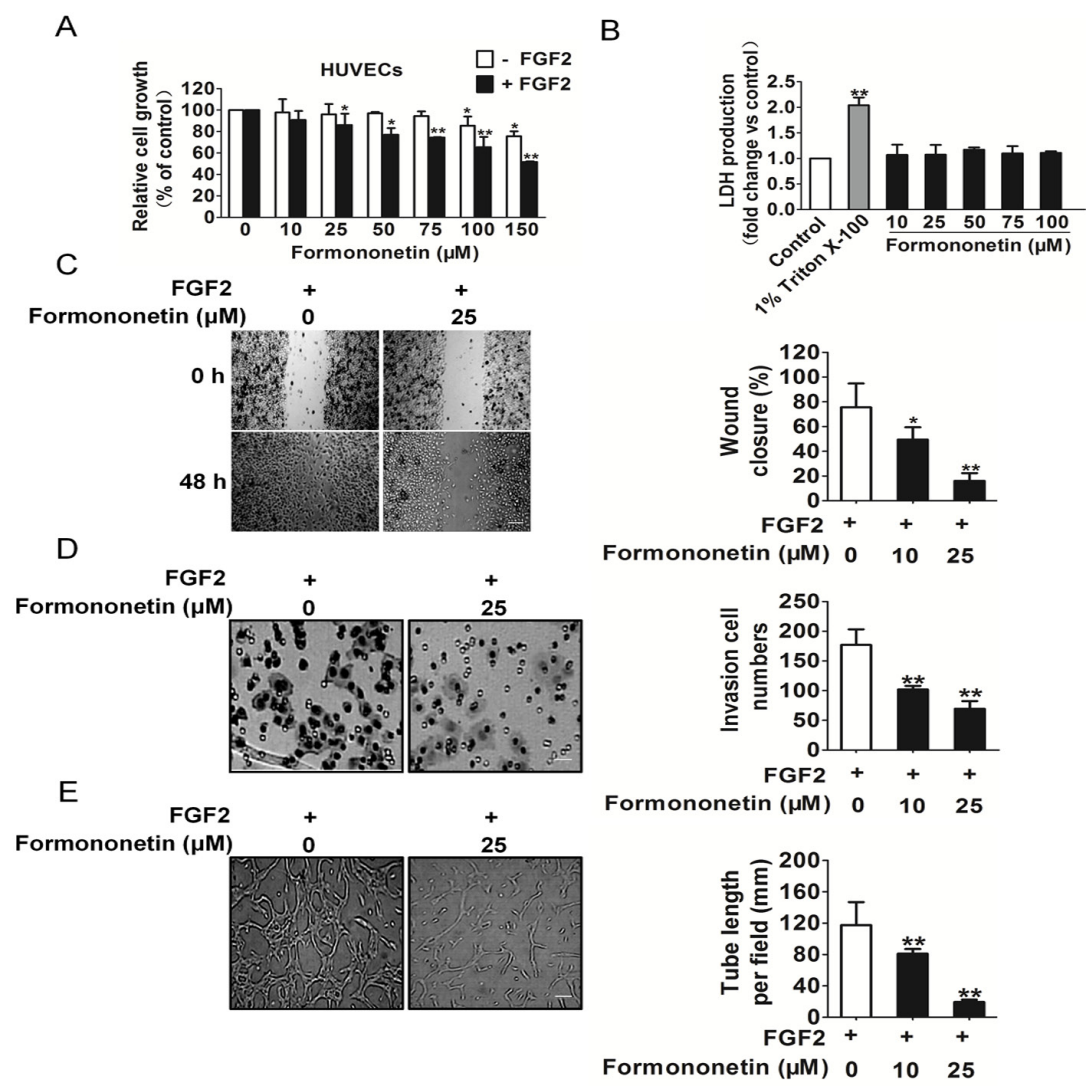
Effects of formononetin on HUVECs proliferation, migration and invasion **A.** The proliferation of HUVECs stimulated by FGF2 was significantly decreased by formononetin in a dose-dependent manner, while formononetin had little inhibitory effects on HUVECs that were not stimulated by FGF2. **B.** Formononetin administration did not result in LDH release, indicating formononetin brought little toxic effects on HUVECs (data are presented as means ± SD, *n* = 6, **P < 0.01 versus control). **C.** Effects of formononetin on HUVECs cell migration in wound migration assays (Scale bar represents 100 μm). **D.** Formononetin decreased the number of invasive cells in a dose-dependent manner (Scale bar represents 50 μm). **E.** Formononetin could dose dependently suppress the capillary lengths of FGF2 stimulated HUVECs (Scale bar represents 50 μm). Data are presented as means ± SD, *n* = 6, *P < 0.05, **P < 0.01 versus FGF2 alone treatment.

To examine the anti-angiogenesis effects of formononetin *in vitro*, proliferation of FGF2 induced HUVECs was detected first. As shown in Figure 2A, the proliferation of endothelial cells stimulated by FGF2 was markedly decreased after formononetin treatment ranging from 25 to 150 μM. Besides, formononetin had obscure inhibition effect on the proliferation of HUVECs in the absence of FGF2. To validate whether formononetin would result in toxicity effects on HUVECs, LDH cytotoxicity assay was carried out. As shown in Figure 2B, Triton X-100 significantly increased LDH release and formononetin brought little toxic effects on HUVECs when compared to vehicle control.

Cell migration and invasion are essential for HUVECs in angiogenesis. We performed wound healing assay (Figure 2C) to investigate the effects of formononetin on cell mobility and observed formononetin strongly inhibited the migration of HUVECs stimulated by FGF2. We also performed transwell invasion assay to evaluate the ability of HUVECs to pass through the Matrigel in the presence of various concentrations of formononetin. As shown in Figure 2D, formononetin significantly inhibited the invasion activities of HUVECs stimulated by FGF2 in a concentration-dependent manner. To elucidate the possible mechanisms of angiogenesis inhibition, tube formation ability of endothelial cells, which is a critical step in the process of angiogenesis, was assessed in HUVECs *in vitro*. As shown in Figure 2E, HUVECs pated on the surface of Migtrigel formed capillary-like structures in the vehicle group within 6 hours. However, treatment with designed concentrations of formononetin strongly inhibited the tube formation of HUVECs.

### Formononetin inhibited FGF2 induce angiogenesis *in vitro* and *in vivo*

**Figure 3:**
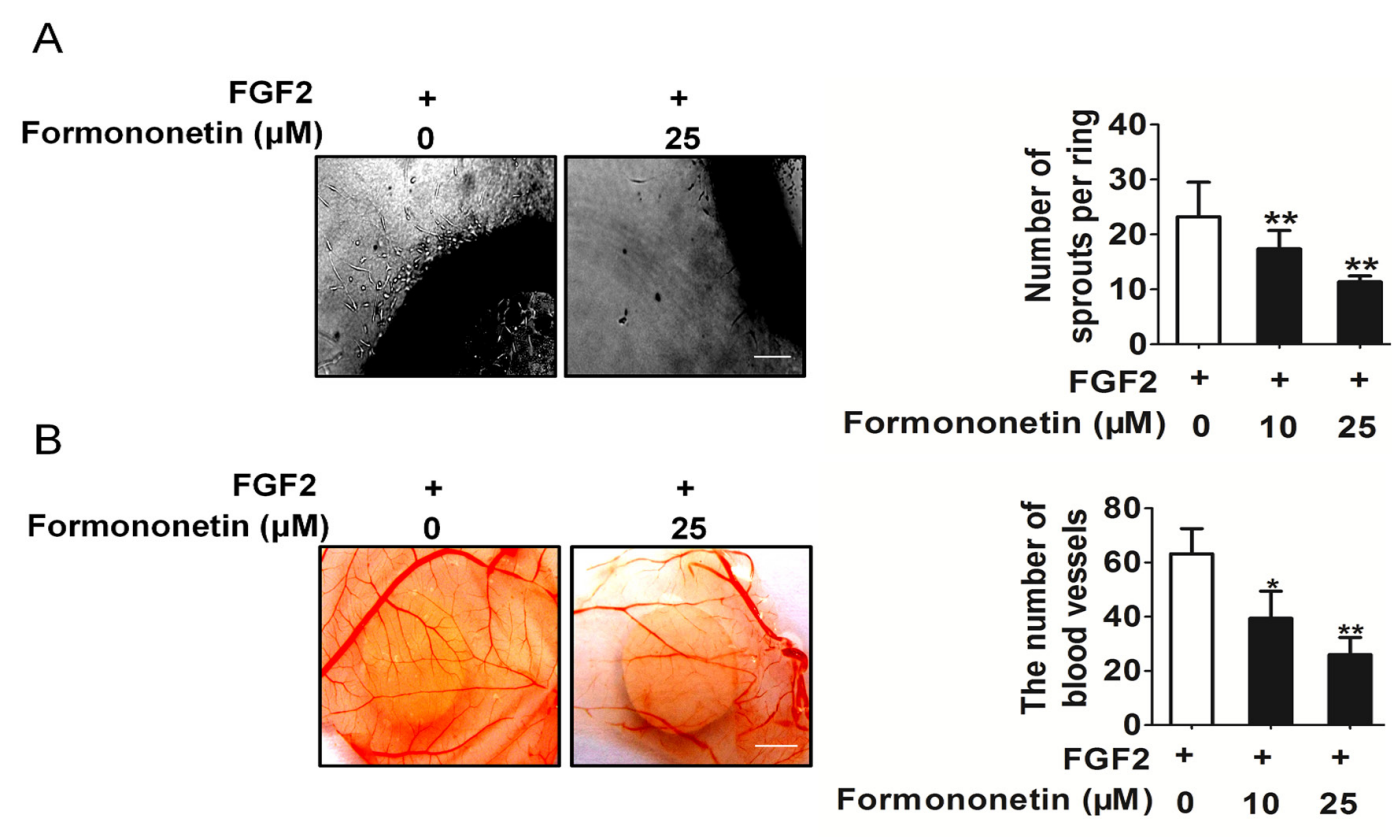
Formononetin inhibits FGF2 induces angiogenesis *in vitro* and *in vivo* **A.** Formononetin dose dependently suppressed sprout formation on the organotypic model of rat aortic ring. Scale bar represents 1 cm. **B.** CAM assay. Photopictographs of a typical experiment showing the angiogenesis pattern in different treatments. Scale bar represents 1 mm. Data are presented as means ± SD, *n* = 3, **P < 0.01 versus FGF2 alone treatment.

To future evaluate the potential effect of formononetin on angiogenesis, two well-established angiogenesis models chicken CAM and rat artic ring assay were used *ex vivo* and *in vivo*. We determined the effects of formononetin on microvessel sprouting *ex vivo* using the rat aortic ring assay. Our results showed that formononetin almost completely inhibited FGF2 induced sprouting from the aortic rings (Figure 3A). Furthermore, in the CAM assay, FGF2 could significantly induce neovascularization, whereas treatment with formononetin potently inhibited FGF2 induced neovascularization (Figure 3B).

### Formononetin inhibited FGFR2 activity in HUVECs

**Figure 4:**
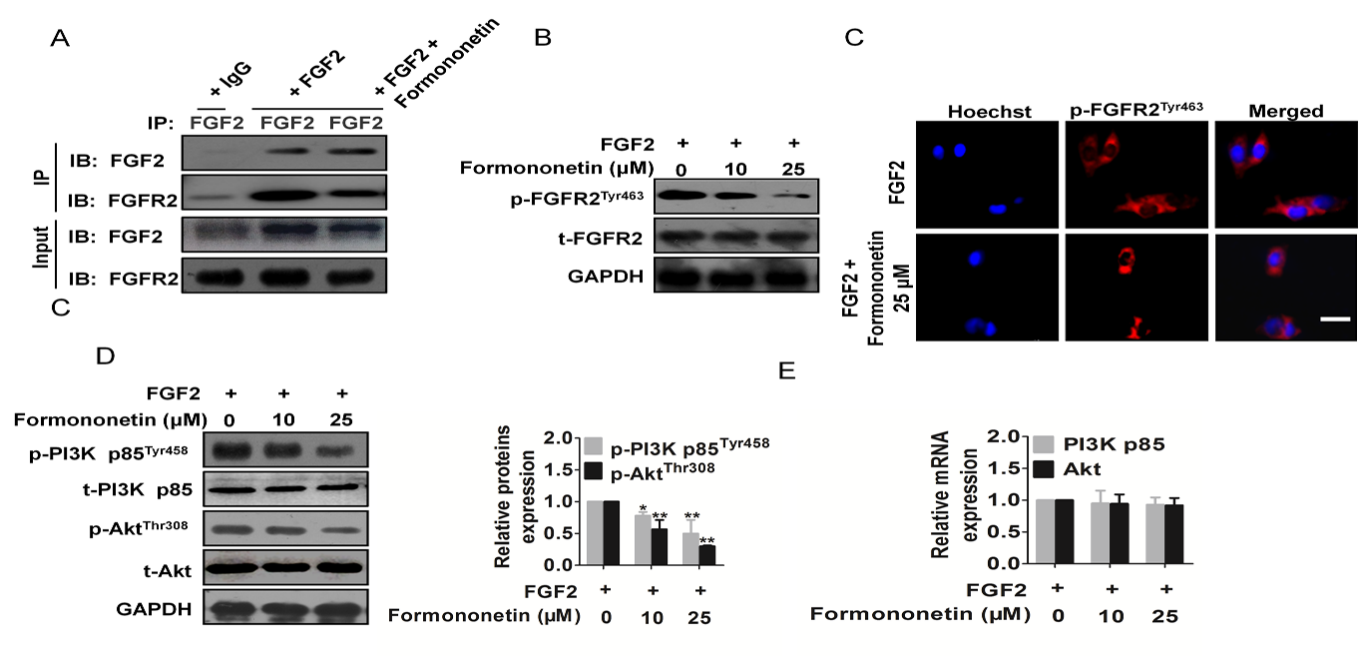
Formononetin attenuated FGFR2 activity and FGFR2 signaling pathway **A.** Immunoprecipitation-western blot analysis using HUVECs revealed that formononetin appeared to decrease FGF2 binding to FGFR2. **B.** Formononetin inhibited FGFR2 phosphorylation in vitro assayed by Western blotting. **C.** Formononetin suppressed the activation of FGFR2 triggered by FGF2 in HUVECs (Scale bar represents 50 μm). **D.** Formononetin inhibited FGFR2 downstream signaling pathway PI3K-Akt in HUVECs. Blots are representative of three experiments. Each has the expression of GAPDH as internal control. **E.** PI3K-Akt mRNA expression in HUVECs treated by formononetin. Data are from three independent experiments and are mean ± SD.

In the presence or absence of extracellular FGF2, the expression of its receptors FGFR2 and FGFR1 on HUVECs remains unchanged (Supplementary Figure 1). However, the phosphorylation of FGFR2 after binding with FGF2 and its downstream protein kinase stimulates angiogenesis. To investigate whether formononetin decreased FGF2 binding to FGFR2, we performed *in vitro* Immunoprecipitation-western blot analysis using HUVEC revealed that formononetin appeared to decrease FGF2 binding to its receptor, FGFR2 (Figure 4A). The same method was applied to assess FGF2 binding to FGFR1 and formononetin effect (Supplementary Figure 2). To verify the Immunoprecipitation-western blot results, we determined the binding of formononetin for FGFR2 using molecular modelling studies (Supplementary Figure 3). Then, we investigated the effects of formononetin on FGFR2 signaling pathway in HUVECs. As shown in Figure 4B and 4C, formononetin clearly reduced FGF2 stimulated of FGFR2 phosphorylation rather than inhibited FGFR1 activity (Supplementary Figure 4). Formononetin also clearly reduced FGF2 stimulated phosphorylation of FGFR2 downstream PI3K and Akt in HUVECs in a concentration-dependent manner (Figure 4D). In contrast, total levels of PI3K, and Akt were not affected by formononetin treatment. Meanwhile, PI3K and Akt mRNA were not inhibited by formononetin (Figure 4E). The above results revealed that formononetin inhibited *in vitro* angiogenesis by directly targeting FGF2-FGFR2 axis on the surface of HUVECs, and further suppressing FGFR2 associated signaling pathways.

### Formononetin inhibited FGF2 stimulated transcriptional activity of STAT3

**Figure 5:**
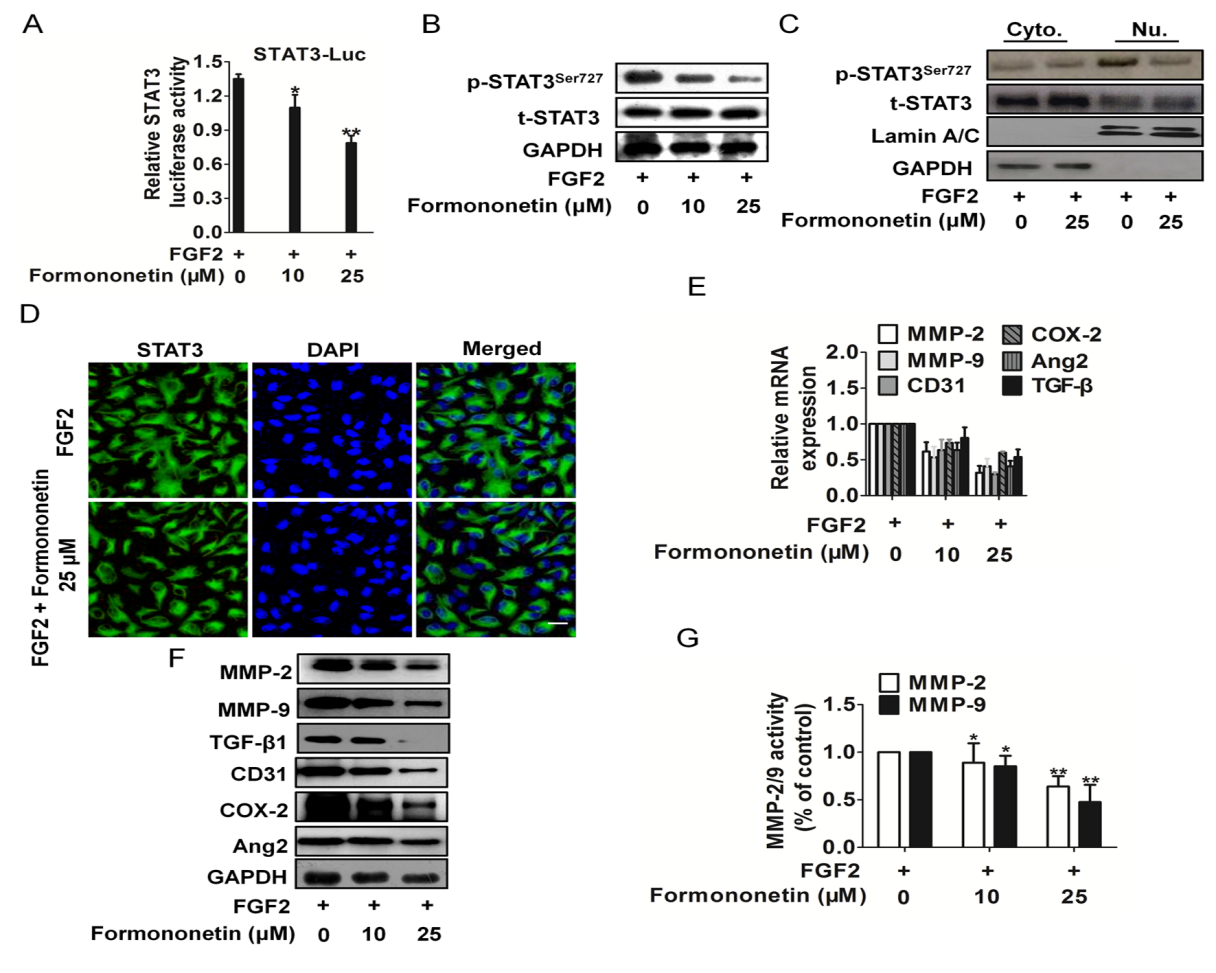
Formononetin attenuates FGF2 induced STAT3 activation in HUVECs **A.** HUVECs grown to 70-90% confluence were co-transfected with p-STAT3-TA-Luc and renilla luciferase (0.1 μg plasmid DNA per well in total) for 18 h, then were sitimulated with FGF2 plus formononetin for 6 h. The cell lysates were performed by DLR assay, and the ratio of firefly luciferase to Renilla (relative luciferase) activity was determined. For indicated comparisons, *P < 0.05, **P < 0.01. **B.** Effect of formononetin on STAT3 protein expression stimulated by FGF2. HUVEC cells were treated with various concentrations of formononetin under FGF2. STAT3 protein expression was analyzed by western blots. **C.** After treated with formononetin, cytoplasmic (Cyto.) and nuclear (Nu.) extracts were prepared and then subjected to western blot for measuring protein level of phosphor-STAT3^Tyr705^ and total-STAT3 respectively. **D.** Immunofluorescentstaining analysis of the effect of formononetin on intracellular STAT3 expression in HUVECs. Cells were treated with formononetin under FGF2. Green color was detected for STAT3, while nuclei were counterstained with blue color using DAPI (scale bar represents 50 μm). **E.** Effect of formononetin on STAT3 target gene mRNA level under FGF2. HUVECs cells were treated with various concentrations of formononetin under FGF2, and protein mRNA level were detected by real-time PCR. **F.** HUVECs were exposed to formononetin in the presence of FGF2. Then, the protein expression was analyzed by western blots. **G.** Quantification of MMP-2/9 activity in HUVECs transfected with formononetin in the presence of FGF2. Bars are represented as the mean ± SD. *n* = 3, **P < 0.01 versus FGF2 treated with FGF2 alone.

To identify transcription factors that are targeted by formononetin, we assessed its effect on the transcriptional activity of several transcription factors that play important roles in HUVECs proliferation and migration. Interestingly, formononetin strongly suppressed FGF2 stimulated STAT3 activity (Figure 5A) rather than c-Jun and NF-κB p65 (Supplementary Figure 5). To further investigate the signaling pathway that mediated STAT3 expression, we assessed the expression of total and phosphorylated STAT3 in HUVECs treated with formononetin by western blotting. We found that the STAT3 total expression level remained unchanged after formononetin treatment, whereas STAT3 phosphorylation was inhibited by formononetin (Figure 5B). However, the effect of FGF2 stimulated activity of c-Jun and NF-κB p65 was not inhibited by formononetin (Supplementary Figure 6). Additionally, the activity of STAT3 was also regulated by subcellular localization, we therefore attempted to explore the effect of formononetin in different cell fractions. The experiment showed that the nuclear STAT3 phosphorylation in HUVEC was evidently abrogated by formononetin (Figure 5C). The activity of STAT3 was also regulated by subcellular localization, we therefore attempted to explore the subcellular distribution of STAT3 by immunofluorescence staining and confocal microscopy. As illustrated in Figure 5D, HUVECs cells treatment with formononetin (25 μM) under FGF2 hardly decreased broad nuclear translocation of STAT3, while FGF2 rendered STAT3 stability and general nuclear distribution. To further investigate whether formononetin can repress the transcription of STAT3 downstream targets which are association with HUVEC migration and invasion, we performed quantitative real-time PCR (qRT-PCR) analyses for STAT3 target genes such as MMP-2/9, transforming growth factor-β1 (TGF-β1), CD31, COX-2, and Ang2. qRT-PCR analyses demonstrated that formononetin decreased the mRNA expression levels of the STAT3 target genes (Figure 5E). In agreement with qRT-PCR results, formononetin also significantly diminished the protein levels of those genes (Figure 5F). These findings demonstrate that formononetin suppresses of STAT3 target gene transcription.

Taking into account that MMPs such as MMP-2 and MMP-9 can be involved in the development of several human malignancies, as degradation of collagen IV in basement membrane and extracellular matrix facilitates tumor progression, including invasion, metastasis, and angiogenesis, we analyzed their activity. Quantification of MMP-2 and MMP-9 activities using a fluorogenic assay showed a significantly decrease in extracellular MMP-2 and MMP-9 activity in formononetin treated HUVECs (Figure 5G).

### Formononetin inhibited cell proliferation and FGFR2 signaling in breast cancer

**Figure 6:**
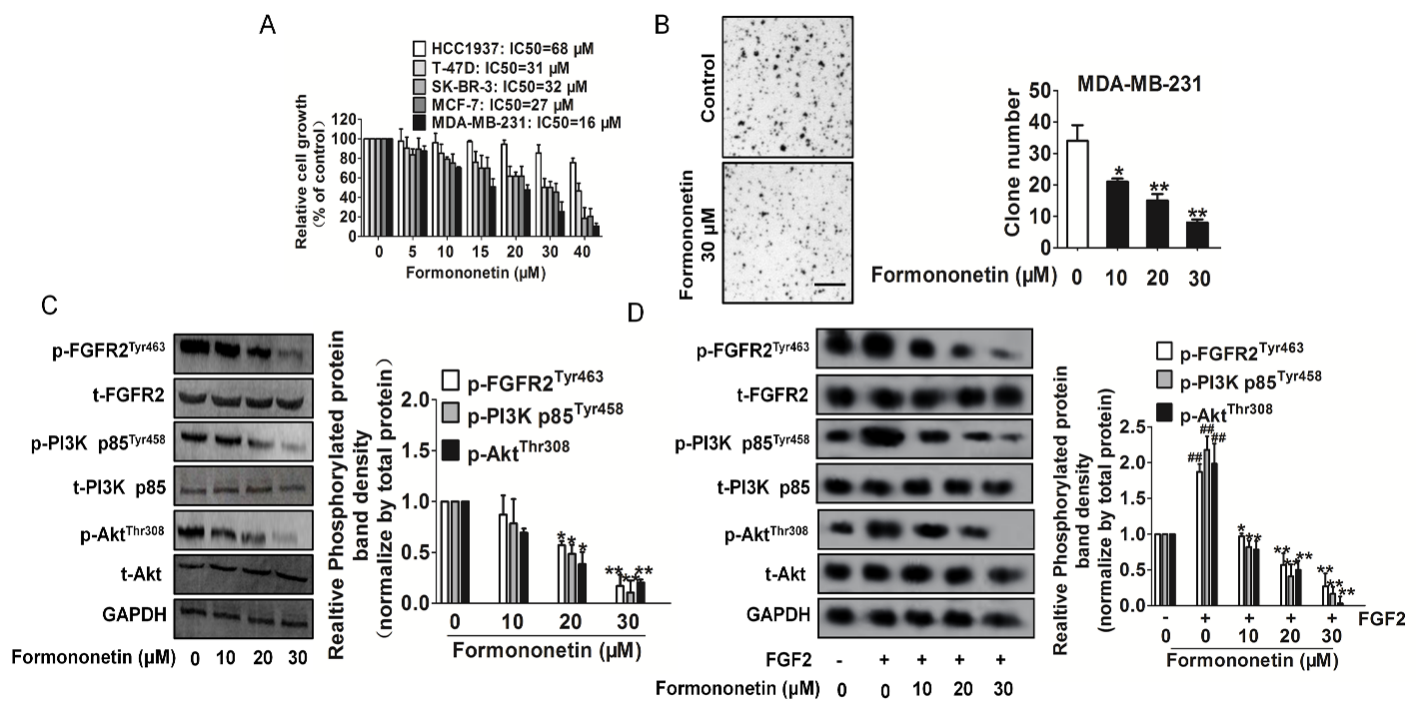
Inhibitory effects of formononetin on tumor cells **A.** Breast cancer cells were exposed to indicated concentrations of formononetin for 24 h. Cell viability was determined by One solution cell proliferation assay. The data are presented as mean ± SD. The values are expressed as percentage of viable cells normalized to percentage of viable cells in 0.5% DMSO-treated cells. **B.** Formononetin inhibited anchorage-independent growth of MDA-MB-231 cells. MDA-MB-231 cells were grown for 3 weeks in 0.25% agarose gel containing vehicle or formononetin. The number of colonies lager than 2 mm in diameter was counted and data represent the means ± SD. of three independent experiments, each performed in duplicate. *P < 0.05, *P < 0.01 vs. vehicle. Scale bars: 1 mm. **C.** Formononetin inhibited FGFR2 downstream signaling molecules, including p-PI3K/PI3K and p-Akt/Akt in a dose-dependent manner. Blots are representative of three experiments. Each has the expression of GAPDH as internal control. Bars are represented as the mean ± SD, *n* = 5, **P < 0.01 versus untreated cells. **D.** Formononetin inhibited FGFR2 downstream signaling molecules stimulated by FGF2. Blots are representative of three experiments. Each has the expression of GAPDH as internal control. Bars are represented as the mean ± SD, *n* = 5, ^**^P < 0.01 versus untreated cells, ^##^ *P* < 0.01 versus FGF2 treated cells.

To access the anti-breast cancer activities of formononetin, four human breast cancer cell lines T-47D, SK-BR-3, MCF-7 and MDA-MB-231, as well as human mammary gland cells Hs 578Bst were chose. All breast cancer cells secrete high mount of FGF2 as well as express higher FGFR2 protein than FGFR1, and the level is much higher than in Hs 578Bst cells (Supplementary Figure 7). As shown in Figure 6A, we found formononetin inhibited breast cancer cell proliferation in a dose responsive manner. IC50 values from each cell line were calculated and we noted the inhibitory effect on Hs 578Bst kept at high micro-molar concentrations than the effect of equivalent doses of formononetin in breast cancer cells. However, the sensitivity of four breast cancer cell lines to formononetin was independent on FGFR2 level based on Figure 6A and Supplementary Figure 7. We also investigated the effect of formononetin on the apoptosis of MDA-MB-231 and MCF-7 cells by DNA fragmentation assay and PARP cleavage assay. Inconsistent with growth inhibition effect, formononetin had no effect on the apoptosis in both MDA-MB-231 and MCF-7 cells (Supplementary Figure 8). Meanwhile, the expression of cleavage PARP was slight in MDA-MB-231 and MCF-7 cells treated with formononetin (Supplementary Figure 8). Collectively, these data demonstrated that formononetin had universal anticancer activity in breast cancer cells and especially inhibited MDA-MB-231 cells proliferation.

To verify whether formononetin could inhibit anchorage-independent growth of MDA-MB-231 cells, we performed soft agar colony formation assays. Formononetin greatly decreased, in a dose-dependent manner, the number and the size of colonies of MDA-MB-231cells grown in soft agar. (Figure 6B), suggesting that formononetin inhibits the *in vitro* transformation capacity of MDA-MB-231 cells. As PI3K and Akt are reported downstream signalings of FGFR2 and also involving in tumor growth, we detected the PI3K and Akt by western blot and RT-PCR. The results showed that the PI3K and Akt activities were significantly reduced after formononetin administration (Figure 6C). In order to confirm formononetin targets FGFR2, we future assayed the FGFR2-PI3K-Akt signaling in the presence of FGF2. As shown in Figure 6D, formononetin significantly suppress FGFR2 phosphorylation and its down-regulate PI3k, Akt activities. Nevertheless, FGFR1 and its downstream regulatory proteins on breast cancer cells were not affected by formononetin (Supplementary Figure 9).

### Formononetin inhibited breast cancer growth and angiogenesis *in vivo*

**Figure 7:**
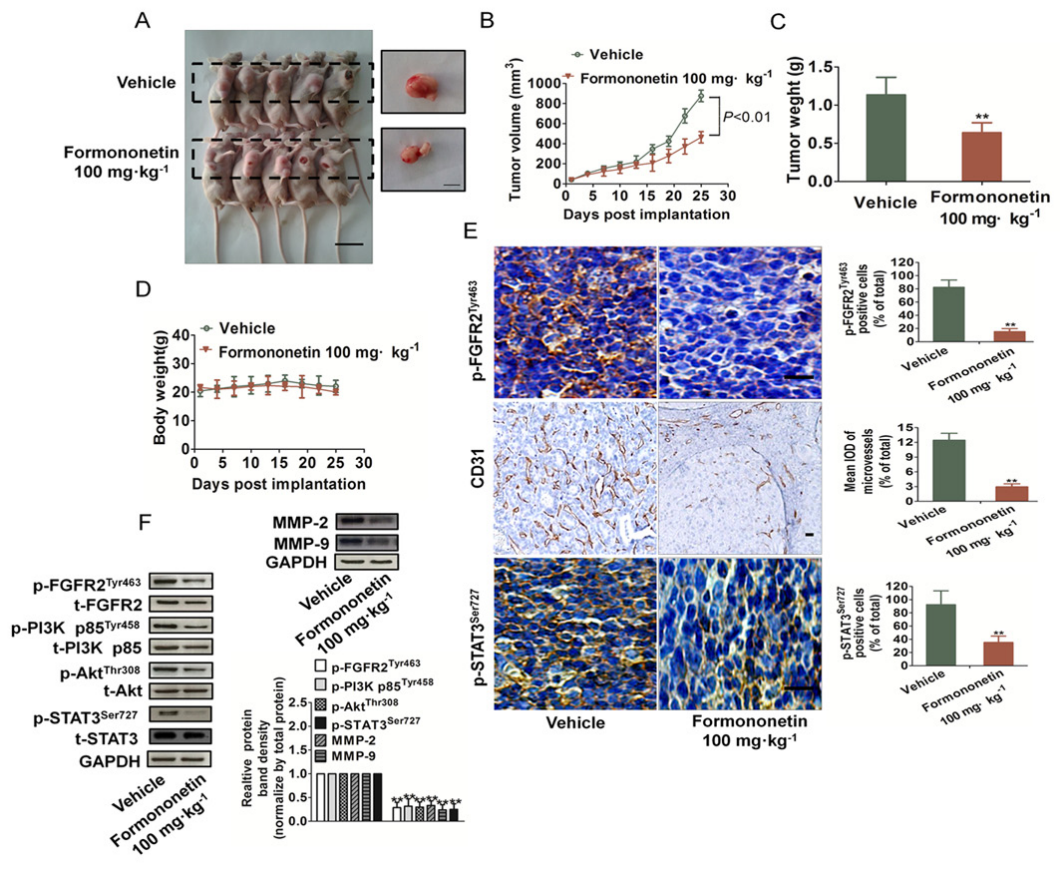
Formononetin inhibited growth and angiogenesis on MDA-MB-231 breast cancer xenografts **A.** Representative mice with MDA-MB-231 xenografts and tumor masses. Scale bars: 1 cm. **B.**-**C.** Treatment with formononetin resulted in significantly tumor growth inhibition versus vehicle-treated control mice. Values represent means ± SD, *n* = 6, **P < 0.01 versus vehicle group. **D.** Body weight changes in formononetin and vehicle treated mice. There was no significant difference in body weight between formononetin and vehicle treated group. **E.** Tumor tissues were prepared for immunohistochemistry detection with antibodies against p-FGFR2^Tyr463^, p-STAT3^Ser727^ and CD31. The statistical results of positive cells and microvessels on the right (Data are presented as means ± SD, *n* = 3, *P < 0.05, **P < 0.01 versus vehicle group). Scale bar represents 50 μm. **F.** Western blot showed the down-regulation of PI3K, Akt, and STAT3 phosphorylation in formononetin-treated group. Down-regulation of MMP-2 and MMP-9 was also observed in formononetin treated group. Data are from three independent experiments and are mean ± SD. *n* = 3, **P < 0.01 compared with control.

To test the anti-angiogenesis effects of formononetin *in vivo*, we utilized breast cancer xenograft model to evaluate whether formononetin could suppress tumor-induced angiogenesis. Prior studies demonstrated that MDA-MB-231 cell line was the often first choice as pre-clinical models for selection of targeted therapies owing to its high aggressive nature either *in vitro* or *in vivo*. Thus, immunodeficient mice bearing MDA-MB-231 xenografts were treated daily with or without formononetin (100 mg/kg) by intragastric administration for 25 days. After treated for 25 d, the mice were sacrificed and tumor tissues were taken out for further analysis. Representative mice with MDA-MB-231 xenografts and tumor masses were shown in Figure 7A. It was found that formononetin dramatically suppressed tumor volumes (Figure 7B) and the formononetin-treated group tumor weight were significantly inhibited compared with the vehicle group (Figure 7C). Furthermore, formononetin treatment was well tolerated, and there was no significant difference in weight between vehicle group and formononetin treated groups (Figure 7D). In addition, no lesion was observed in the heart, liver, spleen, lung, kidney and brain of formononetin-treated mice (data not shown), suggesting that formononetin treatment was well tolerated.

To further examine whether formononetin could suppress breast cancer growth by inhibiting angiogenesis, tumor tissues were stained with specific antibodies against CD31, p-STAT3^Ser727^ and p-FGFR2^Tyr463^. Cluster of differentiation (CD31) is a widely used endothelial marker for quantifying angiogenesis by calculating microvessel density (MVD). Tumor sections stained with anti-CD31 antibody revealed that formononetin inhibited MVD (Figure 7E). Formononetin-treated mice also showed a significant reduction of p-FGFR2^Tyr463^-positive cells in tumors. As shown in Figure 7E, formononetin also decreased phosphorylation of STAT3 in MDA-MB-231 xenograft tumors, which was consistent with the results *in vitro*. In addition, formononetin treatment also resulted in down-regulation of FGF2Rα downstream molecules phosphorylation including PI3K, Akt, STAT3, and MMP-2/9 (Figure 7F). All the results demonstrated that formononetin played an important role in suppressing angiogenesis at least partly through FGF2/FGFR2 signaling pathways.

### Combination of formononetin and VEGFR2 inhibitor sunitinib synergistically blocks tumor growth

**Figure 8:**
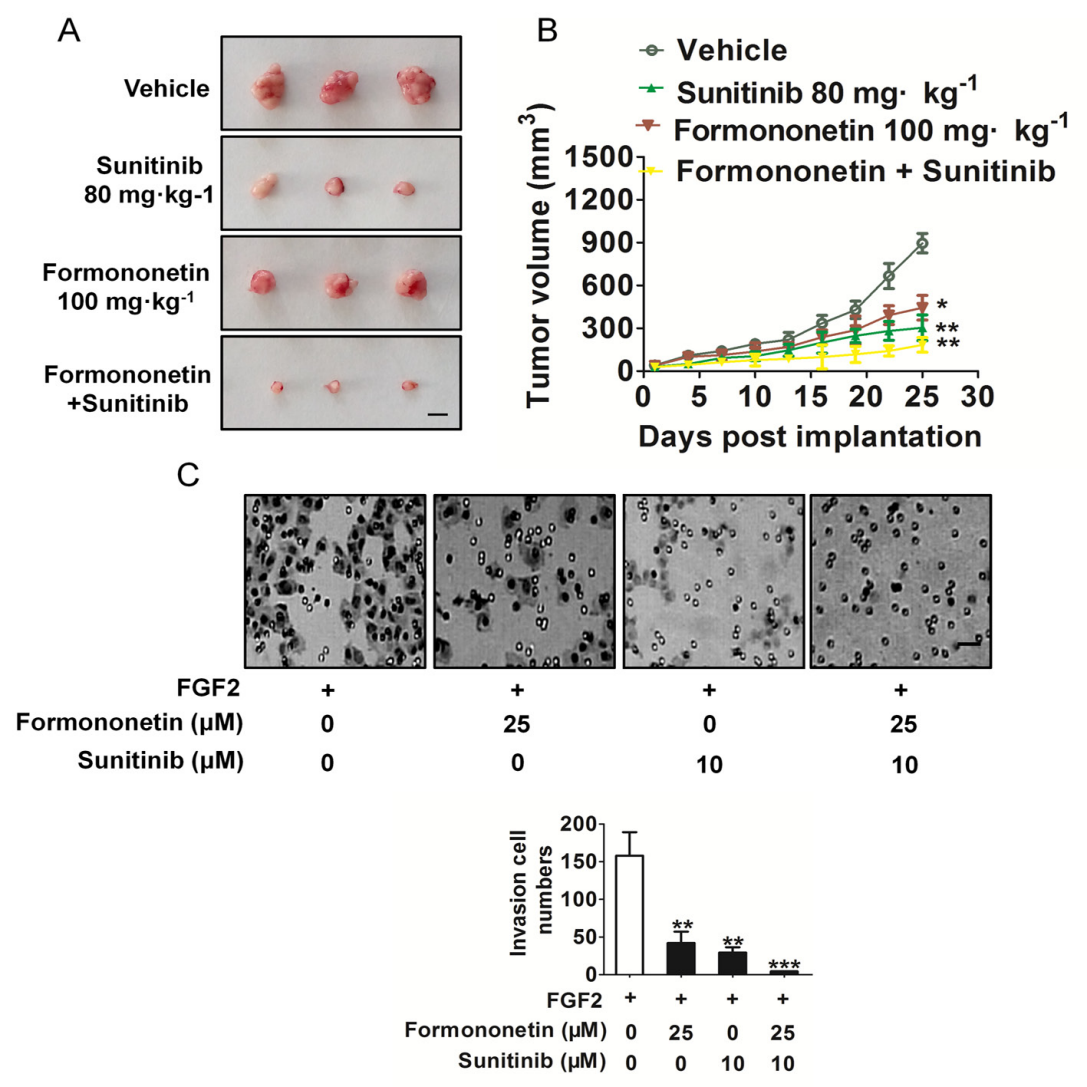
The anti-tumor effect of combination treatment **A.** Mice with appropriate size of tumors were divided randomly into four groups including vehicle-treated group, formononetin dosage group (100 mg/kg/day), sunitinib (80 mg/kg/day) and combination of two. Tumor volume and mice body weight were measured every 3 days. Tumor volume was calculated as mm^3^ = 0.5 × length (mm)^3^ width (mm)^2^. (Values represent means ± SD, *n* = 6, **P < 0.01 versus vehicle group). Scale bar represents 0.5 cm. B, Combination treatment completely inhibited the number of invasive HUVECs. Data are presented as means ± SD, *n* = 6, **P < 0.01, ***P < 0.001 versus FGF2 alone treatment. Scale bar represents 50 μm.

On the basis of the potent *in vitro* and *in vivo* anti-angiogenic and anti-cancer growth properties elicited by formononetin, we next examined if formononetin could enhance the anti-cancer growth efficacy of sunitinib, a VEGFR2 inhibitor. Formononetin and sunitinib alone significantly inhibited tumor tissues compared with the vehicle group, and the combination of these two was significantly more effective (Figure 8A). To address the effects of combination treatment of formononetin and sunitinib on FGF2-induces HUVECs invasion, cells were treated with 25 μM formononetin, 10 μM sunitinib, or their combination. As shown in Figure 8B, the combination treatment almost completely decreased HUVECs invasion *in vitro*.

## DISCUSSION

Tumor angiogenesis is pivotal for tumor growth and metastasis. Among the numerous factors involved in angiogenesis, the role of VEGF and VEGFR2 is well established, but other angiogenic factors switch on during cancer progression and induce resistance to VEGFR inhibitors monotherapy [2]. FGF2 is a pleiotropic cytokine that stimulates endothelial cell growth, migration, and survival. Unlike VEGF, FGF2 selectively binds its receptor FGFR2 and stimulates angiogenesis, tumor growth and endothelial cell migration, indicating that FGF2 has biological activity *in vivo* [27]. FGFR2 activity has previously been shown to act through the PI3K/Akt signaling pathway. Our group has been engaged in the screening novel angiogenesis inhibitors and in this study, we identified formononetin had significant inhibitory effects on HUVECs function by suppressing HUVECs proliferation, migration, invasion, and tube formation *in vitro*. HUVECs proliferation plays an important role in the process of angiogenesis from preexisting vessels, therefore, we examined whether formononetin showed anti-proliferative effects on HUVECs after stimulation by FGF2. The results showed that formononetin markedly inhibited HUVECs proliferation stimulated by FGF2. Angiogenesis are complex processes which occur by a series of complex events including endothelial cells migration and invasion. In this study, according to wound healing, Transwell invasion assays, formononetin effectively inhibited the migration and invasion of HUVECs. Furthermore, we have demonstrated that formononetin inhibits FGF2-dependentstimulation of the capillary-like networks formation of endothelial cells, as well as suppresses FGF2 induced blood vessel formation in CAM, which was consistent with the result that formononetin could inhibit rat artery ring sprouting.

Protein-protein interactions and regulate the signal transduction circuitry play pivotal roles in tumor metastasis as well as angiogenesis. FGF2 regulates angiogenesis and by binding to its receptor FGF2R, which is expressed in vinous cancer cells, including non-small cell lung cancer (NSCLC), gastric carcinoma and prostatic cancer (Supplementary Figure 10). FGF2R is required for the migration and growth of endothelial cell. The functions of vascular endothelial cells rely on FGF2R signaling and FGF2R phosphorylation initiates downstream signaling pathways [28]. In this study, we identified formononetin disrupt FGF2 interaction with its receptor FGF2R and inhibited FGF2 binding to its receptor FGFR2. Western blot results indicated that formononetin selectively inhibited the activity of FGF2R and immunofluorescence assay verified these results. Activation of the PI3K-Akt pathway has been shown to promote endothelial cells proliferation and motility. Akt regulates several endothelial cells functions such as migration and proliferation, and stimulates the production of STAT3 transcription factors. In this study, formononetin significantly inhibited FGF2 stimulated phosphorylation of FGF2R and downstream PI3K and Akt in HUVECs, indicating its ability to block angiogenesis. STAT3 is a well-known key downstream component of the PI3K-Akt pathway. Phosphorylated STAT3 is translocated into the nucleus to transmit extracellular signals that regulate cell growth, differentiation, proliferation, apoptosis, and migration functions [29]. STAT3 is a well-known inducer of MMPs and angiogenic factors including TGF-β, CD31, COX-2 and Ang2 in tumor cells. We have detected a significant decrease in extracellular MMP-2/9 activity and expression. Moreover, formononetin inhibited the expression of TGF-β, CD31, COX-2 and Ang2 downstream-regulator of STAT3, which stimulates tumor angiogenesis and tumor growth.

**Figure 9:**
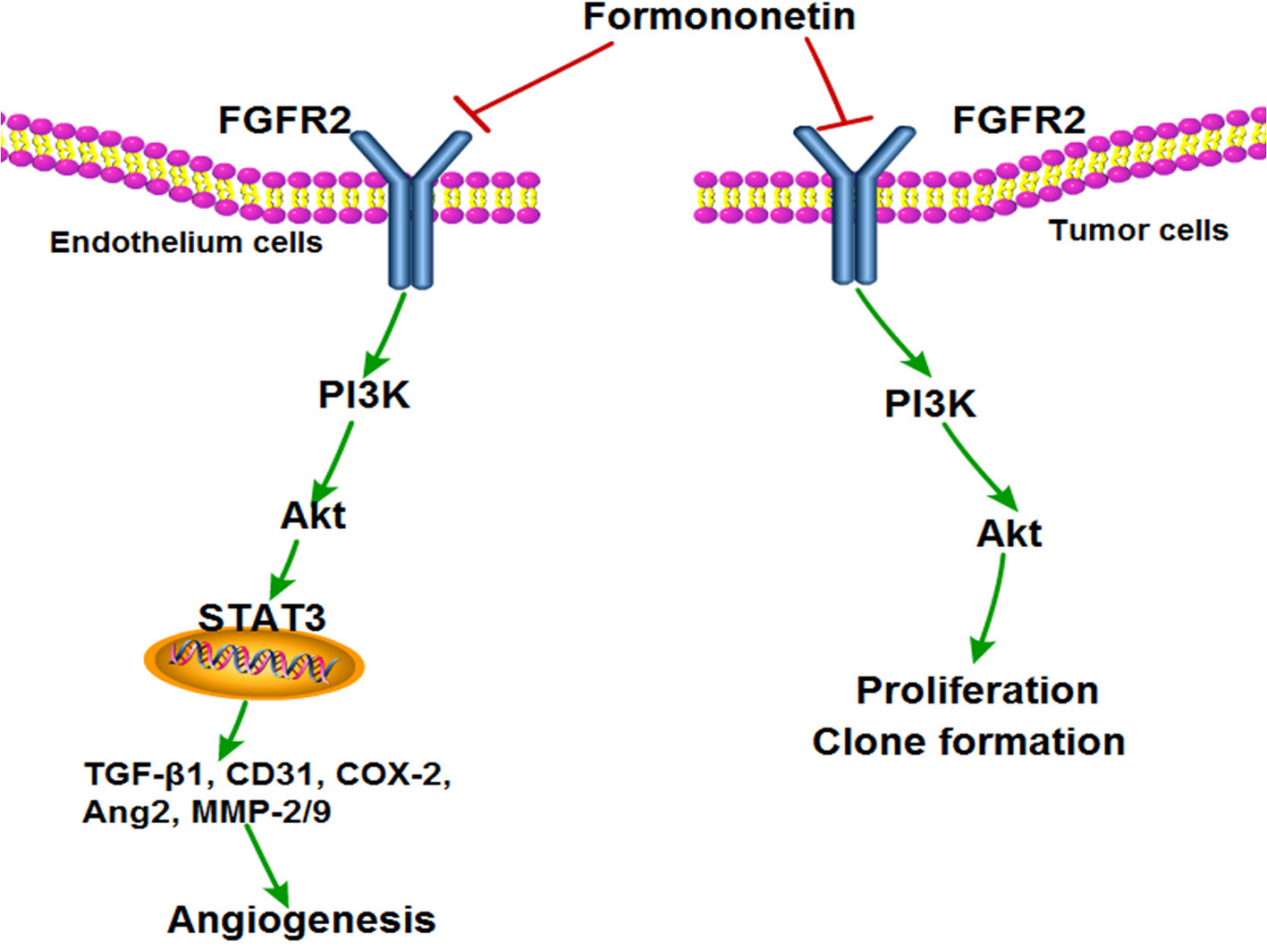
Proposed model by which formononetin treatment suppresses tumor angiogenesis and growth via inhibiting FGFR2 signaling pathway

Besides inhibiting angiogenesis, formononetin also had a direct inhibitory effect on tumor cells. It showed inhibitory effects in cancer cell viability assays and the IC50 values of formononetin inhibited the proliferation of MDA-MBN-231cells at 16 μM, which are most sensitive to formononetin treatment among the cancer cells treated, in a concentration-dependent manner. Extending these analyses *in vivo*, formononetin obviously inhibited tumor angiogenesis *in vivo* in a nude mouse xenograft model where human breast cancer cells were grown subcutaneous. Histological studies of tumor sections revealed that formononetin significantly reduced angiogenesis indexed by CD31 and p-FGFR2^Tyr463^ antibodies. Moreover, similar to the FGF2 signaling inhibition effects observed *in vitro*, formononetin also significant decrease PI3K, Akt, MMP-2, MMP-9, and STAT3 in MDA-MB-231 tumor sections according to western blot, further demonstrating that formononetin played an important role in suppressing angiogenesis at least in part via FGFR2 signaling pathway. For another experiment *in vivo*, the combination treatment caused much more tumor suppression than that of single treatment of either formononetin or sunitinib, which further confirmed our conclusion. Consistently, the combination treatment significantly inhibit HUVECs invasion, which is an important step in the tumor angiogenesis process. Taken together, these results demonstrate that a novel molecule capable of disrupting the binding of FGF2 to its receptor FGFR2 and inhibits FGFR2-dependent signaling and suppresses angiogenesis and tumorigenesis (Figure 9).

## MATERIALS AND METHODS

### Cell culture and formononetin preparation

The human breast cancer cell line T-47D, SK-BR-3, MCF-7 MDA-MB-231 andhuman mammary gland epithelial cells HCC1937 was purchased from the ATCC, and maintained in L-15 medium supplemented with 10% FBS. HUVEC was purchased from Chi Scientific, and were cultivated in gelatinized culture plates in M199 medium supplemented with 15% FBS, 1% PS, 50 μg/ml endothelial cell growth supplement (ECGS, BD Bioscience) and 100 μg/ml heparin. Formononetin (98%, Sigma-Aldrich, St. Louis, MO) was dissolved in dimethyl sulfoxide (DMSO, final concentration is 0.1%) to prepare required concentrations.

### One solution cell proliferation assay

The cell viability was determined by CellTiter 96^®^ Aqueous One Solution cell proliferation assay (Promega, Madison, WI, USA). Briefly, cells were seeded in 96-well cell culture plates and treated with indicated agents. After incubation for indicated time period, 20 μL of One Solution reagent were added to each well and incubation was continued for additional 4 h. The absorbance was measured at 490 nm using Synergy™ HT Multi-Mode Microplate Reader (Bio-Tek, Winooski, VT, USA). The effect of indicated agents on cell viability was assessed as the percent of cell viability compared with vehicle-treated control cells, which were arbitrarily assigned 100% viability [16]. The concentration of formononetin resulting in 50% inhibition of control growth (IC50) was calculated by SPSS statistics software.

### Lactate dehydrogenase (LDH) toxicity assay

The LDH released into cell cultures is an index of cytotoxicity and evaluation of the permeability of cell membrane. HUVECs were seeded in 96-well plate at a density of 3 × 10^3^ cells per well. After incubation with vehicle (0.1% DMSO), 1% Triton X-100 or various concentrations of formononetin for 24 h, cell supernatants were collected and analyzed for LDH activity using LDH cyto-toxicity assay kit from Keygen biotech. The absorbance of formed formazan was read at 490 nm on a microplate reader [16].

### Wound healing

We examined the migration of HUVECs using a wound-healing assay. Briefly, cells were each grown on 3.5-cm plates with their respective culture media. After the growing cell layers had reached confluence, we inflicted a uniform wound in each plate using a pipette tip, and washed the wounded layers with PBS to remove all cell debris. Then, we evaluated the closure at 48 h using bright-field microscopy [17].

### Invasion assay

Assay was performed with Matrigel-coated chambers from a BioCoat Matrigel Invasion Chamber Kit (BD Biosciences). Cells with 500 μl in serum-free medium were added into the upper chamber and complete medium was added into the lower chamber. After incubation for 24 h, non-invasive cells in the upper surface of the membrane were removed and the cells invasion to the lower surface of the membrane were fixed. Cell counting was then carried out by photographing the membrane through the microscope [18] and five random fields were taken.

### Anchorage-independent growth assay

Soft agar colony-formation assays were performed as previously described with minor modifications [19]. MDA-MB-231 (1 × 10^4^) cells in 1.5 mL of growth medium were mixed with 1.5 mL of 0.5% agarose in warmed growth medium containing vehicle (0.1% DMSO) or formononetin and layered on 0.5% base agar in 60-mm cell culture dishes. Culture medium containing scoparone was added only once; subsequently, medium without formononetin was added every week for 21 days until large colonies were evident. Cells were stained with crystal violet for colony counting.

### Tube formation assay

The tube formation assay was performed using 12-well plate coated with 100 μl Matrigel basement membrane matrix (BD Bioscience) per well and polymerized at 37°C for 30 min. HUVECs suspended in M199 medium containing 2% FBS were plated on the Matrigel at a density of 2 × 10^5^ cells/well. Formononetin (10, and 25 μM) were then added together with FGF2. After 6 h, The Matrigel-induced morphological changes were photographed and the extent of capillary tube formation was evaluated by measuring the total tube length per field [20].

### Rat aortic ring assay

Rat aortic ring assay was performed as described previously [21]. In brief, 48-well plates were coated with 120 μL of Matrigel per well and polymerized in an incubator. Aortas isolated from 6-week-old male Sprague-Dawley rats were cleaned of periadventitial fat and connective tissues in cold phosphate-buffered saline and cut into rings of 1~1.5 mm in circumference. The aortic rings were randomized into wells and sealed with a 100 μL overlay of Matrigel. FGF2 in 500 μL of serum-free M199 with or without formononetin was added into the wells, and the fresh medium was exchanged for every 2 d. After 6 d, microvessel sprouting was fixed and photographed using an inverted microscope (Olympus).

### Chick chorioallantoic membrane assay

Chick chorioallantoic membrane (CAM) assay was performed, as described previously [22].

### Western blotting assay

In brief, cell lysates were separated by 8% SDS-PAGE and transferred to polyvinylidene difluoride membranes. Membranes were then incubated with primary antibodies including indicated antibodies. After overnight incubation at 4°C, membranes were incubated with secondary antibodies. Immunoreactive bands were then visualized by the enhanced chemiluminescence (ECL) detection system (GE healthcare).

### FGFR2 kinase inhibition assay

The IC50 values for inhibition of FGFR2 by formononetin was determined using a FRET-based *in vitro* kinase assay (Z'-lyte assay, Invitrogen, Paisley, UK). The kinase domains of FGFR2 was assayed in 50 mm HEPES pH 7.5, 0.01% BRIJ-35, 10 mm MgCl2, 2 mm MnCl2, 1 mm EGTA, 1 mm DTT, with 20 μm or 80 μm ATP, respectively. The assay was performed in triplicate in 384-well plates according to the manufacturer's instructions [23].

### Immunofluorescence analysis

The effects of formononetin on FGF2 induced expression of FGFR2 phosphorylation in HUVECs were examined using an immunocytochemical method [24]. Cells were pretreated with or without formononetin for 24 h in the presence of FGF2. For immunofluorescent labeling, anti- p-FGFR2^Tyr463^ antibody was used as primary antibody and goat anti-rabbit IgG-FITC was used as a secondary antibody. Fluorescence cells were observed and photographed under a laser scanning confocal microscope (LEICA TCS SP5, Mannheim, Germany).

### Immunoprecipitation assay

HUVECs were lysed in a culture dish by adding 0.5 mL of ice-cold RIPA lysis buffer. The supernatants were collected by centrifugation at 15,000 *g* for 10 minutes at 4°C and then incubated with IgG or FGF2 in presence or absence of formononetin at 4°C overnight, followed by incubation with anti-FGF2 for 4 hours. Then, supernatants were incubation with protein G-Sepharose (Santa Cruz) for 4 hours. Following the removal of supernatant by brief centrifugation (6,000 g), the protein G-Sepharose were washed 3 times with lysis buffer and then boiled for 5 minutes in loading buffer [16]. Immunoprecipitates was further analyzed by western blotting using anti-FGFR2 antibody and anti-FGF antibody.

### Luciferase reporter gene assay

HUVECs in 96 well culture plates were transiently transfected with 0.1 μg/well p-STAT3-TA-Luc reporter plasmids, p-c-Jun-TA-Luc reporter plasmids or p-NF-κB p65-TA-Luc reporter plasmids (Biotime Biotech, Haimen, China). Transfection efficiency was normalized with renilla luciferase reporter plasmids. After 18 h post-transfection, cells were treated with indicated agents. Relative promoter activity was measured by dual-luciferase reporter (DLR) assay system using the Glomax 96 Microplate Luminometer (Promega, Madison, WI, USA).

### Quantitative real-time PCR (qRT-PCR) analysis

HUVECs cells were incubated with formononetin for 24 h. Total RNA was extracted using the QIAzol lysis reagent (Qiagen, Valencia, CA, USA), and 1 μg of total RNA was reverse transcribed using the RevertAid First Strand cDNA synthesis kit (Thermo Scientific, Waltham, MA, USA) according to the manufacturers' instructions. For qRT-PCR, 25-50 ng of cDNA was used for PCR amplification using Power SYBR Green PCR Master Mix (Applied Biosystems, Warrington, UK) with the ViiA™ 7 Real-Time PCR System (Applied Biosystems). Acidic ribosomal phosphoprotein P0 (RPLP0; 36B4) was used as an internal control [25]. PCR condition and primer sequences are listed in Supplementary Table 1.

### Xenograft models and immunohistochemistry detections

3×10^6^ human breast cancer MDA-MB-231 cells were subcutaneously implanted into female, BALB/c nude mice to build breast cancer xenograft. Mice with appropriate size of tumors were divided randomly into two groups including vehicle-treated group and Formononetin dosage group (100 mg/kg/day). The mice were treated with formononetin or carboxy methylated cellulose (vehicle) daily by intragastric administration. Tumor volume and mice body weight were measured every 3 days. Tumor volume was calculated as mm^3^ = 0.5 × length (mm)^3^ width (mm)^2^ [26]. After sacrificing mice on day 25, tumors and normal tissues will be harvested for western blotting. Band intensities were quantified using image-J software. Deparaffinized tumor sections were stained with specific antibodies including CD31, p-STAT3^Ser727^, and p-VEGFR2^Tyr951^. Detection was done with avidin-biotin-HRP complex (Thermo scientific) and diaminobenzidine as chromogen. Nuclei were counterstained with hematoxylin. p-FGFR2^Tyr463^ positive cells were counted in five random high-power fields per section and were reported as a percentage of positive cells in each cellular compartment. Mean integrated optical density (mean IOD) of blood vessels accords to the following formula: mean IOD=IOD/area of the tumor section. All animal experiments were carried out in compliance with the Guidelines for the Shandong University School of Medicine.

### Statistical analysis

The data were presented as mean ± SD. Differences in the results of two groups were evaluated using either two-tailed Student's t test or one-way ANOVA followed by post-hoc Dunnett's test. The differences with *P* < 0.05 were considered statistically significant.

## SUPPLEMENTARY MATERIAL FIGURES AND TABLE


