# Supplementary material for: Formononetin, a novel FGFR2 inhibitor, potently inhibits angiogenesis and tumor growth in preclinical models
Source: Oncotarget. 2015 Nov 12;6(42):44563–78. doi: 10.18632/oncotarget.6310 (PMC4792576; doi:10.18632/oncotarget.6310)
Supplement: Supplementary file 1 [file oncotarget-06-44563-s001.pdf]

Formononetin, a novel FGFR2 inhibitor, potently inhibits angiogenesis and tumor growth in preclinical models

Supplementary Materials

Molecular docking methods

The molecular docking studies were carried out using GOLD 5.0 (Genetic Optimization of Ligand Docking, The Cambridge Crystallographic Data Centre (CCDC), Cambridge, UK). The crystal structure of FGFR2 (PDB ID: 1GJO) was retrieved from the RCSB Protein Data Bank and chosen as the structure of the reference protein. An 8 Å sphere around the centroid of the ligand was used to define the active site region. The pre-process of FGFR2 was carried out using Discovery Studio 2.55 (Accelrys, Inc., San Diego, CA, USA) software package by adding hydrogen atoms, including water removal and assigning Chemistry at HARvard Macromolecular Mechanics. Formononetin was also built and its geometry was optimized in Discovery Studio 2.55. The docking scheme was modified as described previously [1].

ELISA

Cells (7×10^5) were plated in six-well dishes and treated for 24 h with formononetin, or vehicle. Supernatants were collected and ELISA for FGF2 was performed with FGF2 (Human) ELISA Kit (KA0527; Abnova) following the manufacturer's instructions.

Apoptosis assays

Apoptosis assays were tested in MCF-7 and MDA-MB-231 cell lines with or without formononetin using Apoptosis Detection kit I (BD Biosciences, USA) and C6 Flow Cytometer (USA).

References

[1] Frederick Cohen, Philippe Bergeron, Elizabeth Blackwood, Krista K. Bowman, Huifen Chen, Antonio G. DiPasquale, Jennifer A. Epler, Michael F. T. Koehler, Kevin Lau, Cristina Lewis, Lichuan Liu, Cuong Q. Ly, Shiva Malek, et al. Potent, selective, and orally bioavailable inhibitors of mammalian target of rapamycin (mTOR) kinase based on a quaternary substituted dihydrofuropyrimidine. J Med Chem 2011; 54: 3426-3435.

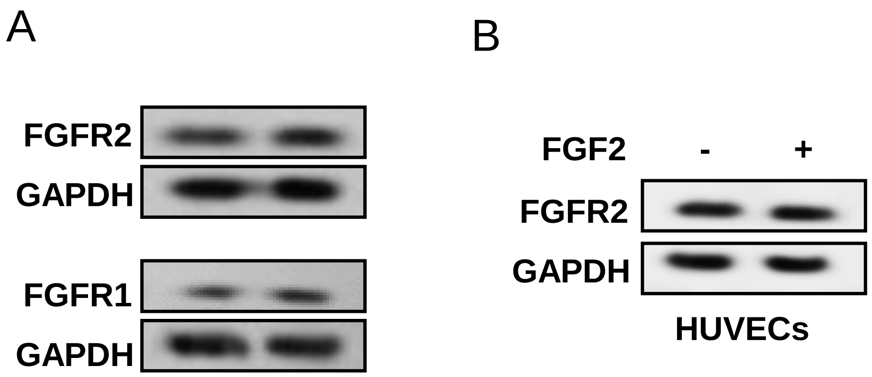

Supplementary Figure 1. A. The expression level of FGFR1 is low than FGFR2 in HUVECs. **B.** Western blotting analysis shown protein expression of FGFR2 was independent on FGF2 in HUVECs.

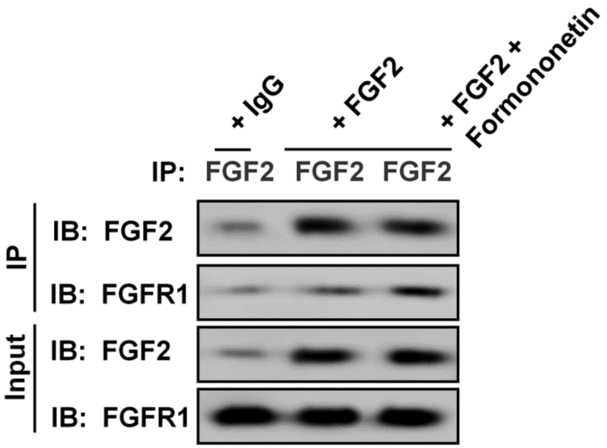

Supplementary Figure 2. Immunoprecipitation-western blot analysis using HUVECs revealed that FGF2 binding to FGFR1 was not blocked by formononetin.

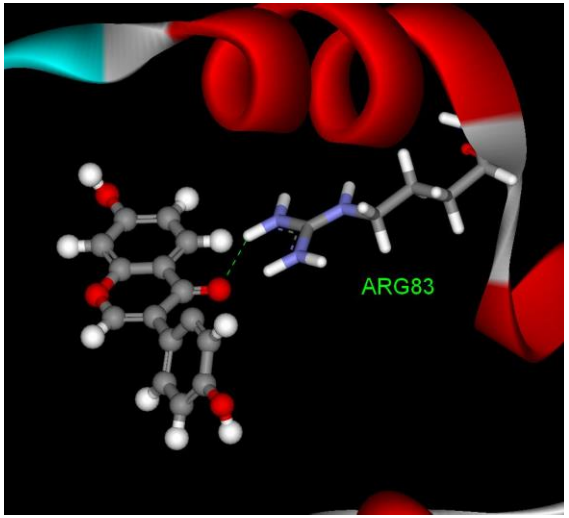

Supplementary Figure 3. The interaction modes of formononetin with the kinase domain of FGFR2 (PDB entry 1GJO) by computer simulation and computer-based molecular docking methods. Formononetin was docked into the active site of FGFR2 and the interactions between formononetin and FGFR2 are shown in the 3-D structure. The interaction was the hydrogen bond formed between formononetin and FGFR2. The hydrogen bond was between the carbonyl and ARG83.

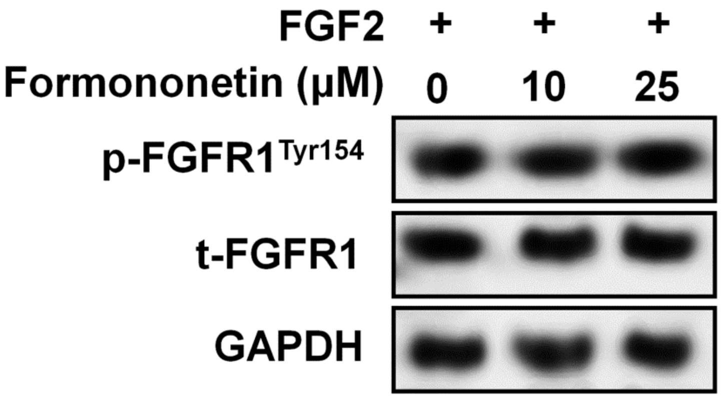

Supplementary Figure 4. The effect of formononetin on FGFR1 phosphorylation in vitro assayed by western blotting assay.

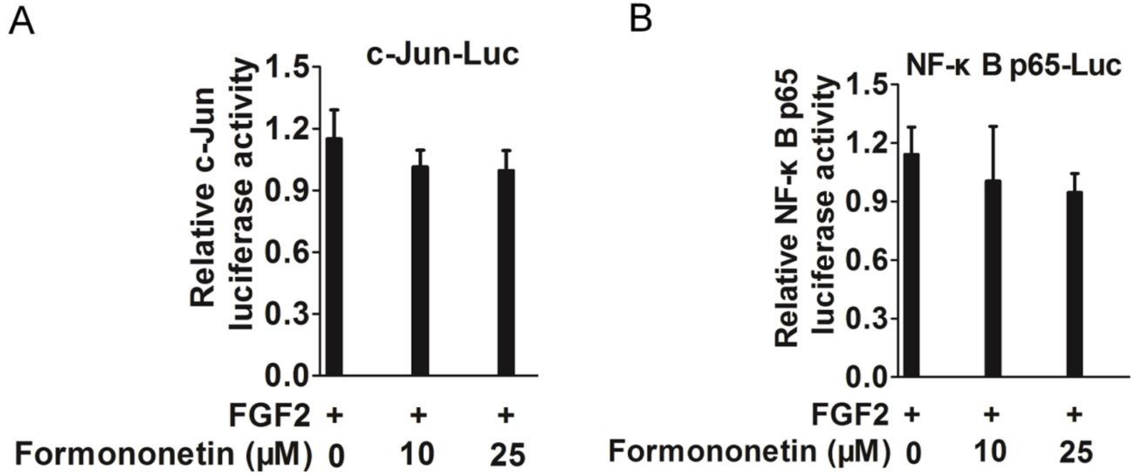

Supplementary Figure 5. The effect of formononetin on the activities of transcriptional factor NF-κB p65 and c-Jun. ***A-B,*** HUVECs grown to 70-90% confluence were co-transfected with p-c-Jun-TA-Luc or p-NF-κB p65-TA-Luc and renilla luciferase for 18 h, then were sitimulated with FGF2 plus formononetin for 6 h. The cell lysates were performed by DLR assay, and the ratio of firefly luciferase to Renilla (relative luciferase) activity was determined.

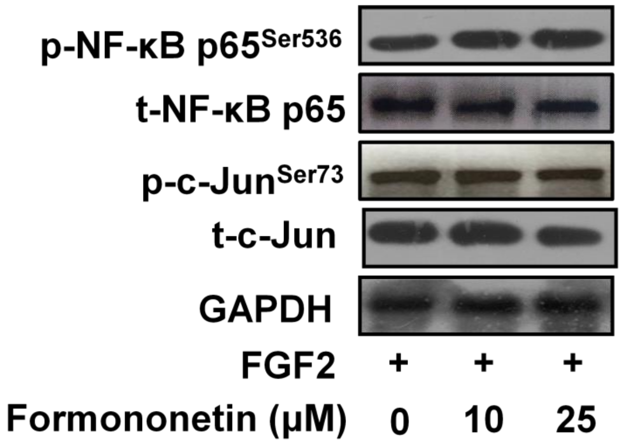

Supplementary Figure 6. The effect of formononetin on FGF2-stimulated activity of c-Jun and NF-κB p65. p-NF-κB $p65^{Ser536}$, t- NF-κB p65, p-c-Jun^{Ser73} and t-c-Jun were measured in HUVECs cell after formononetin treated in the presence of FGF2. GAPDH expression was used as a loading control.

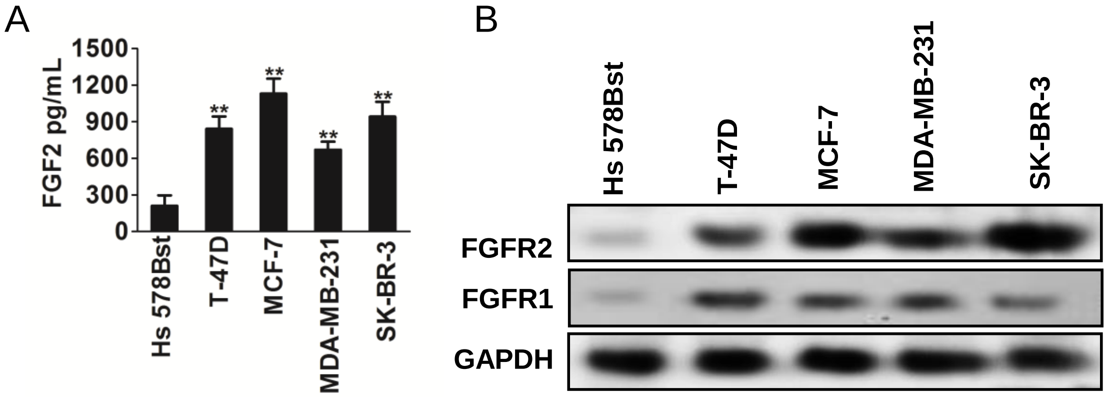

Supplementary Figure 7. A. Quantification of secreted FGF2 in breast cancer cell lines T-47D, SK-BR-3, MCF-7 and MDA-MB-231, as well as human mammary gland cells Hs 578Bst by ELISA. Data are from three independent experiments and are mean ± SD. n=3, ** $P < 0.01$ compared with Hs 578Bst cells. **B.** Biochemical analysis of FGFR2 and FGFR1 in protein extract of T-47D, SK-BR-3, MCF-7, MDA-MB-231 and Hs 578Bst cells. Protein loading was normalized by GAPDH.

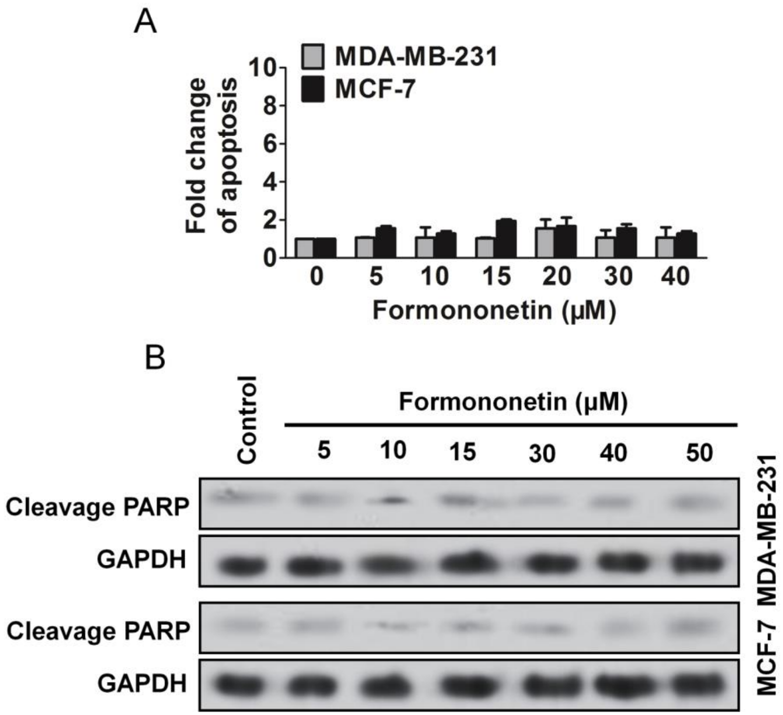

Supplementary Figure 8. A. Cell apoptosis in MDA-MB-231 and MCF-7 cells treatment with formononetin. Data were collected from three independent experiments and were average ± SD. values. B. Western blot analysis showed that cleaved PARP was slight in both control cells and cells treated with formononetin.

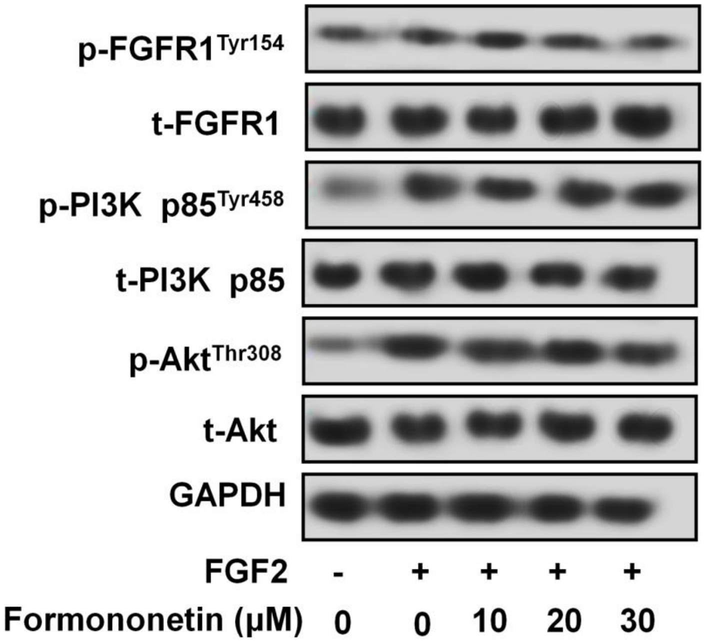

Supplementary Figure 9. The effect of formononetin on FGFR1 activity and FGFR1 signaling pathway.

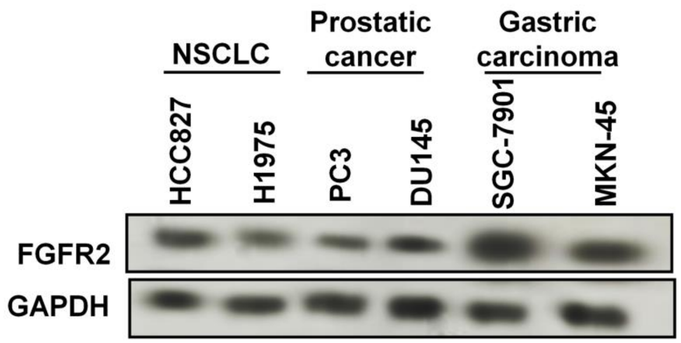

Supplementary Figure 10. The expression of FGFR2 in NSCLC, gastric carcinoma and prostatic cancer cells.

Supplementary Table 1. Primers used for PCR and sequence analysis

PI3K p85	Forward: 5'-GCT CCT GGA AGC CAT TGA GAA-3'
	Reverse: 5'-GT CGA TCA TCT CCA AGT CCA C-3'
Akt	Forward: 5'-GTGGCCAAGG ACGAGGTG-3'
	Reverse: 5'-ACAGGT GGAAGAACAGCT CGC-3'
MMP-2	Forward: 5’- GGCTCATGCCTTCGCCCCAG-3’
	Reverse: 5’- ACTCCCCATCGGCGTTCCCA-3’
MMP-9	Forward: 5’-TGACAGCGACAAGAAGTG-3’
	Reverse: 5’- CAGTGAAGCGGTACATAGG-3’
CD31	Forward:5’-TCAACTTCAAGCTCCTAA-3’
	Reverse: 5’-CCACTCAGACTTTATTCAAA-3’
COX-2	Forward:5’-TCACAGGCTTCCATTGACCAG-3’
	Reverse: 5’-CCGAGGCTTTTCTACCAGA-3’
Ang2	Forward:5’-TGCTGGAGAACATTCTAGAGAAC-3’
	Reverse: 5’-CACAGTCTCTGAAGGTGGTTT-3’
TGF-β1	Forward: 5’-ACCATGCCGCCCTCCGGG-3’
	Reverse: 5’-TCAGCTGCACTTGCAGGAGC-3’
β-actin	Forward: 5′-GCTGCGTGTGGCCCCTGAG-3′
	Reverse: 5′-ACGCAGGATGGCATGAGGGA-3′
